# THE EXPERIENCES OF COLORECTAL CANCER SURGERY AMONG OLDER PATIENTS WHO LIVED ALONE IN TAIWAN

**DOI:** 10.1093/geroni/igae098.2435

**Published:** 2024-12-31

**Authors:** Li-Ying Chen, Tsuann Kuo

**Affiliations:** Taipei City Hospital Renai Branch, Taipei, Taipei, Taiwan (Republic of China); Chung Shan Medical University, Taichung, Taichung, Taiwan (Republic of China)

## Abstract

In Taiwan, it is a common practice that family takes care of their older adults when hospitalized. However, people who were never married or living alone would face challenges in coming up with a caregiver in the hospital. The shortage of caregiver among Taiwanese older population was due to its low fertility and high aging rate as Taiwan will enter a super-aged society in 2025. The purpose of this paper was to understand how inpatient surgical experiences on older adults who lived alone. The research methodology used qualitative interviews with structural questions followed by content analysis. Eight older adults participated to share their experiences who live alone and hospitalized for colorectal cancer surgery. The result showed that prognosis, postoperative care and Activities of daily living(ADL) were concerned. Older adults living alone were not only worried about the results of the surgery but also concerned about their reduced self-care ability and postoperative care without help. In addition to determining the recovery of digestive system function, doctors consulted rehabilitation to strengthen their mobility, taking into account the factor of living alone. Based on the results, the author suggested that it is essential to discuss postoperative care arrangements and whether the patient will be able to regain their self-care abilities after surgery. With the increasing number of people living alone, greater emphasis should be placed on the patient’s own decisions not relatives. For older patients living alone and undergoing inpatient surgery, developing in-hospital rehabilitation plans is crucial to strengthen their self-care abilities.

